# Comparison of Three Physical—Cognitive Training Programs in Healthy Older Adults: A Study Protocol for a Monocentric Randomized Trial

**DOI:** 10.3390/brainsci11010066

**Published:** 2021-01-06

**Authors:** Marta Maria Torre, Antoine Langeard, Nicolas Hugues, Jérôme Laurin, Jean-Jacques Temprado

**Affiliations:** 1 Aix-Marseille Université, CNRS, ISM, Institut des Sciences du Mouvement, CEDEX 09, 13007 Marseille, France; antoine.langeard@unicaen.fr (A.L.); nicolas.hugues@univ-amu.fr (N.H.); jean-jacques.temprado@univ-amu.fr (J.-J.T.); 2 Institut National de la Santé et de la Recherche Médicale, U1075 COMETE, 14032 Caen, France; 3 Aix-Marseille Université, INSERM, Institut de Neurobiologie de la Méditerranée, CEDEX 09, 13007 Marseille, France; jerome.laurin@univ-amu.fr

**Keywords:** aging, cognition, cognitive-motor training, Nordic walking, exergames

## Abstract

(1) Combining aerobic, coordination and cognitive training allows for more improved physical and cognitive performance than when performed separately. A Nordic walking (NW) and two cognitive-motor circuit training programs (CT-c and CT-fit) are compared. CT-c and CT-fit stimulate cognition differently: CT-c, is through conventional complex coordination training performed in single and dual-task conditions; CT-fit, incorporates it into complex goal-directed actions, implemented by fitness gaming technology (2) The aim is to determine whether CT-fit brings additional benefits to cognition compared to more traditional training. (3) Forty-five healthy independent living community dwellers participants (65–80 years) will be included after a general medical examination. The main exclusion criteria are signs of cognitive impairments (Mini–Mental State Examination < 26/30) and physical impairments. Pre and post-tests will be performed to assess: cognitive functions (Montreal Cognitive Assessment; Trail Making Test; Stroop task, working memory test, Rey Complex Figure copy task, Oral Trail Making Test, and dual-task); motor fitness (Bipedal and unipedal balance test, gait assessments, Time Up and Go, chair sit and reach test and four-square stepping test); and physical fitness (10 m incremental shuttle walking test, maximal handgrip force, Timed-Stands test). (4) Incorporating cognitive demands into complex, goal-directed actions using fitness gaming technology should be the best solution to optimize training benefits.

## 1. Introduction

Aging leads to significant declines in brain functions, physical and cognitive performance that are responsible for the loss of behavioral adaptability in many daily living tasks. Most of these alterations result from sedentary or insufficiently physically and cognitively engaging behaviors. Consequently, developing effective solutions to counteract and delay these alterations is a major challenge for aging research and society. It is now widely demonstrated that physical activity can contribute to achieving this objective. The benefits of aerobic exercise and muscular resistance training for brain and cognition (e.g., executive control, working memory, and memory functions) have been demonstrated in numerous studies, reviews, and meta-analyses [1,2,3,4,5,6]. Moreover, a recent study showed that combining aerobic and muscular resistance exercise is more effective and beneficial for cognition than the single isolated aerobic or muscular resistance training [7,8]. Recently, however, Diamond and Ling [9] challenged the systematic link between aerobic exercise and executive functions (EF). Their findings highlighted the importance of the qualitative aspects of the motor skills supporting training interventions, together with the quantitative aspects (intensity, duration, frequency of sessions) (see Pesce [10] for a convergent point of view). Specifically, they argued that the magnitude of benefits of aerobic training on cognition depends on the type of motor skill supporting the aerobic effort, that is, whether the physical activity requires a more or less cognition-demanding control of movement (e.g., whole-body or multi-limb coordination). Indeed, several studies reported a positive relationship between older adults’ motor fitness (measured by movement speed, balance, fine coordination, and flexibility) and cognitive performance [11,12]. Evidence of this relationship also comes from interventional studies showing that complex motor skill training improved cognitive performance [13,14]. In addition, a systematic review concluded that coordination training induces a greater improvement of cognitive functions, compared to aerobic or/and muscular resistance training [15]. In older adults, the effectiveness of complex motor skills training to improve cognitive functioning could be reinforced by cognitive-motor dedifferentiation [16,17], which is reflected by increased correlations among cognitive, sensory, and motor domains with age [18,19]. Cognitive-motor dedifferentiation has been confirmed by brain imaging studies. They showed larger activation of the prefrontal areas during motor tasks in older adults, which suggested that executive functions become more involved in complex movement control (e.g., interlimb coordination) compared to younger subjects [20,21]. Convergent evidence also suggested a strong link between age-related alteration of executive functions and deficits in gait, posture [22], and bimanual coordination [23]. According to this theoretical framework, it can be hypothesized that, in older adults, motor skills training involves more cognitive resources than in younger adults and could, therefore, be particularly appropriate to improve cognitive functions [24]. Consequently, it can also be hypothesized that combining cardiovascular (aerobic) and complex motor movement training should be a more effective way to improve cognitive functions [25,26]. This issue was addressed in a previous study [27] by comparing the effects of Nordic walking training (NW), mainly relying on aerobic exercise [28,29], and those of a circuit training program (CT) combining aerobic exercise and complex motor skills. While NW was expected to induce significant benefits on cognition via exercising cardio-respiratory capacities and muscular strength, CT was conceived to allow practicing complex motor skills in combination with aerobic exercise, and then, to capitalize on the coalition of their respective effects on cognition. Accordingly, it was predicted that combining aerobic exercise and complex motor skill training (i.e., during CT) would result in greater effects on cognitive functioning than the NW intervention. However, the results did not confirm this hypothesis, thereby suggesting that NW training was more cognitively demanding than hypothesized in most physiological studies, which considered Nordic walking as only a highly demanding aerobic activity. This could be due to the complex coordinated movements involved in stick manipulation and the constraints on balance control resulting from walking on uneven ground. In addition, in our previous study, motor exercises practiced during the CT were seemingly not optimally designed and progressively incremented in difficulty to impact cognition. Thus, the question emerged of whether increasing the cognitive demands of the motor exercises included in the CT would result in greater benefits to cognitive functions than NW. The present study addresses this issue by relying on the framework proposed by Herold and colleagues [30], who differentiated two types of simultaneous association of cognitive and motor exercise in motor-cognitive training, namely the “Thinking while moving” (TwM) and “Moving while thinking” (MwT) task conditions. TwM training consists of dual-task (DT) training, that is, conditions in which a cognitive task is added to the motor task as a distractor for the cognitive resources, as when walking straight on a line at a given speed to reach a location while counting backward. According to Herold and colleagues, in dual-task training, the cognitive task (i.e., counting backward) is not relevant to the goal-directed walking performance. Instead, it perturbs the performance by forcing the participants either to share their cognitive resources between the two tasks or, if unable to do, to give a priority to one of them or to switch quickly one to the other [31,32]. Nevertheless, DT training has been shown to improve cognitive functions, as well as performance in complex motor-cognitive tasks (e.g., walking in environments strewn with obstacles) [33,34]. Conversely, MwT training refers to situations in which the cognitive processes are incorporated into the motor task, so that they are a prerequisite for the success of the goal-directed motor task. This happens, for instance, when walking as fast as possible around a pre-defined space in order to pick up some numbered objects in a given order (memorized or discovered while walking) or when a decision must be made while moving to find a way or to switch from one movement pattern to another. Thus, in the MwT training conditions, optimal functioning of cognitive processes (visual search, memory, and spatial orientation) is inherently required to succeed in the motor task [30]. Herold and colleagues [30] hypothesized that MwT training could be more effective than other training conditions (i.e., aerobic alone or complex motor tasks performed either in single or dual-task condition) since there are closer to cognitive and motor requirements of real-life situations (see Raichlen and Alexander [35] for a similar point of view). Therefore, capitalizing on our previous study [27], the purpose of the present experiment is to compare the effects on physical and cognitive capacities of the NW training program and those of two different circuit training, based on TwM and MwT task conditions, respectively. In the circuit training based on TwM conditions (henceforth, CT-c), cognitive processes are loaded thanks to the inclusion of complex whole-body and interlimb coordinated movements performed in single or dual-task situations. In the circuit training based on MwT conditions (henceforth, CT-fit), the cognitive processes are directly incorporated into complex goal-directed motor tasks, thanks to the use of a fitness gaming technology (i.e., the Fitlight Trainer™; Fitlight Sports Corp., Canada). This technology is currently used in sports training [36] and rehabilitation to improve physical and cognitive skills (e.g., reaction time, perception-action capacities, agility, and coordination). To our best knowledge, until now, studies that compare these two types of training (or comparable fitness gaming technologies) in older adults are lacking.

### Aims and Hypotheses

The present experiment aims at comparing the effects on cognitive and motor functions of three types of training programs (NW, CT-c, and CT-fit), which differ in the nature of cognitive-motor exercises. It is hypothesized that, while all training would induce similar physical and motor fitness benefits, the CTs protocols would induce higher cognitive benefits than NW. Moreover, CT-fit should induce higher executive benefits than the two other training programs, thanks to the inclusion of cognitive stimulation through MwT conditions.

## 2. Materials and Methods

### 2.1. Study Design

The study is a monocentric randomized trial. Training interventions will be carried out at the “Stade Marseillais Université Club” (SMUC, Marseille, France) that is, a multisport club proposing programs of physical activity adapted to older adults. All subjects must give their informed consent for inclusion before they participate in the study. The study will be conducted in accordance with the Declaration of Helsinki, and the protocol has been approved by the French National Ethics Committee (CPP IDF10 no. 2019-A03263-54) (registered also on ClinicalTrials.gov ID: NCT04504643). Pre- and post-tests will be carried out before and after the training period to evaluate the cognitive, physical, and motor benefits of each program. An evaluation will be done at the Institute of Movement Science (ISM) (Marseille, France).

### 2.2. Recruitment

Participants will be recruited through an advertisement in the local newspaper. After contacting the project manager, either through phone calls or e-mails, volunteers to participate in the study will be screened for inclusion.

### 2.3. Study Population and Eligible Criteria

Participants are independent-living community dwellers aged between 65 and 80 years. General health status and lifestyle behaviors will be assessed via telephone. Primary exclusion criteria are: signs of cognitive impairment, reflected by a Mini-Mental State Examination (MMSE) score below 26/30 [37], inability to practice physical activity at moderate to vigorous intensity, uncontrolled psychiatric or cardiovascular affections, uncorrected hearing and/or a visual impairment, and psychotropic or bradicardizing medical treatments all assessed by medical evaluations and screenings. Participants will be examined for each criterion separately. Participants familiar with the Fitlight Trainer™ methods and or ranked highly active according to the International Physical Activity Questionnaire (IPAQ) [38] will also be excluded. The ability to practice a moderate to vigorous physical activity (i.e., 60–70% of maximal heart rate or HRmax) will be assessed by a medical examination and incremental exercise test on a cycle-ergometer [39] supervised by cardiologists at the sports medicine department of the local hospital of Saint Marguerite in Marseille. The termination criterion of the incremental cycling test is fixed at 85% of the estimated HRmax (220-age). Electrocardiogram will be recorded during exercise. HR will be measured at rest, during the effort, and 3 min post-exercise test. Initial mechanical power will be set at 30 W for 1 min and then the workload will increase each minute by 15 W. Adaptation of the exercise protocol will be considered by medical doctors for participants reporting previous cardiovascular problems (e.g., 30 W/10 W/1 min; 10 W/10 W/1 min). Complete inclusion and exclusion criteria are listed in Table 1.

### 2.4. Randomization

A computer-generated, random sequence of permuted blocks (block size = 3), will be created in order to assign the requested number (15) of screened participants to each group.

### 2.5. Informed Consent

**Figure 1:**
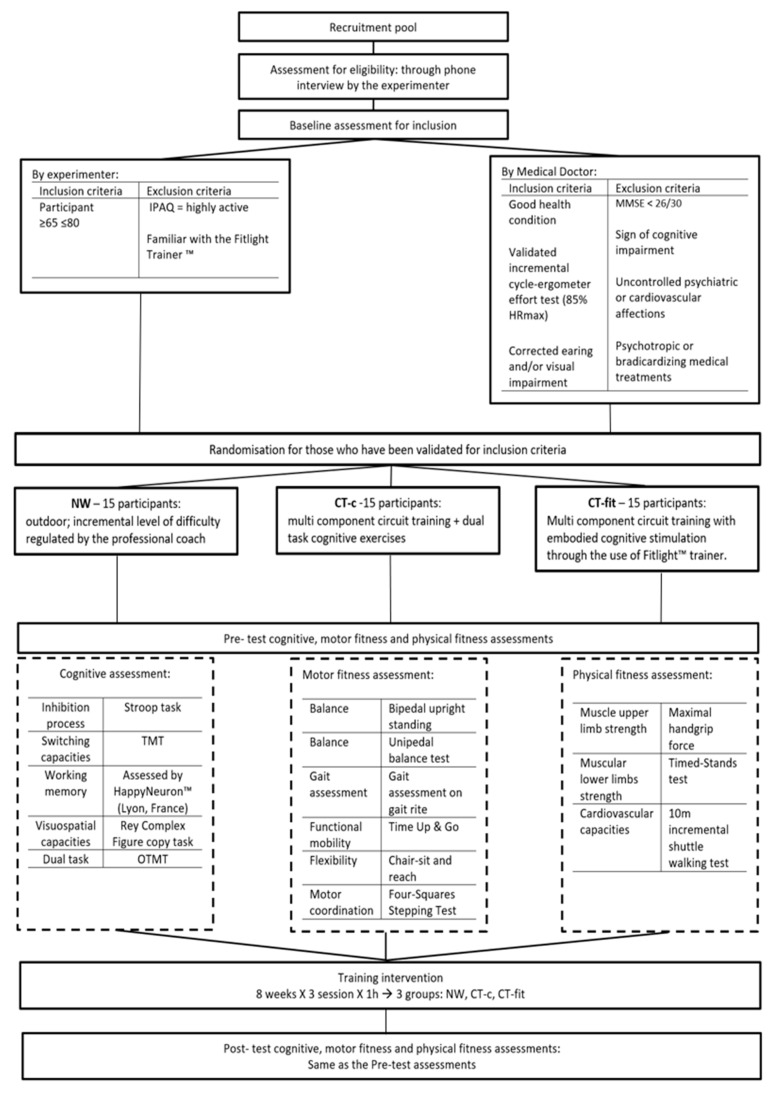
Participant’s flow chart. Participant’s flow chart (IPAQ = International Physical Activity Questionnaire; TMT = Trial Making Test; OTMT = Oral Trial Making Test).

Information about the organization of the training programs (location, schedule, attendance, coaches, evaluations, etc.) will be presented to the eligible participants by the principal investigator. No information will be provided, however, about the research hypotheses. Then, participants will sign an informed consent form. Participants flows are presented in Figure 1.

### 2.6. Intervention Description

The three training programs are administered by two professional coaches, one for NW and one for the two circuit training interventions (CT-c & CT-fit). The three programs last 8 weeks, with 3 sessions of 1-h per week (24 h in total). For each intervention program, core training sessions will last 50 min, while the remaining 10 min are spent equally on the warm-up and cool-down. The heart rate of the participants of the three groups is recorded via a heart rate monitor provided to each of them before starting the training program and pre-set with their characteristics (gender, weight, height). The heart rate monitor allows controlling the intensity level of physical effort during the core session. During each session, participants are encouraged by coaches to maintain an intensity corresponding to 60–70% of HRmax. The heart rate monitor also allows ensuring that the mean heart rates reached during each session are roughly equivalent to the different training programs. In each session, at the end of the core session, just before the cool-down, participants will be asked to rate their effort using the Perceived BORG scale, ranging between 1 and 10 (with 1 representing absolutely no effort and 10 extreme maximal effort) [40].

#### 2.6.1. Nordic Walking (NW)

NW sessions will be performed in a nature park, which offers pathways of different lengths and levels of difficulty. Walking sticks will be provided by the coach to participants. As in our previous study [27], the coach will progressively increase the level of difficulty over the 8 weeks, trying to individualize, as far as possible, the requested effort level while managing the whole group in the nature park. The participants will be following the coach, who shows the way and adapts the walking speed and difficulty.

#### 2.6.2. Conventional Circuit Training (CT-c)

The CT-c protocol will start with a 5 min warm-up for muscle activation and stretching (to avoid articular and muscle injuries); 20 min of aerobic exercises to fully stimulate the aerobic metabolism; 30 min of circuit training divided into different stations (see below the detailed description) to be repeated 2 times; and a 5-min cool-down.

**Figure 2:**
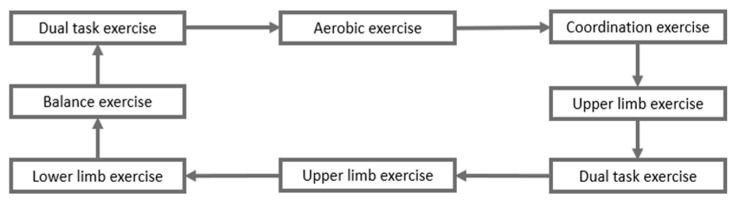
Example of the organization of the CT-c stations.

Circuit training stations will be organized in eight successive training stations dedicated to: (i) cardio-vascular exercises (2 different stations), (ii) muscular resistance exercises (2 different stations), (iii) balance, stepping, interlimb, and whole-body coordination exercises (2 different stations), and (iv) motor-cognitive dual-tasks exercises (2 different stations) (see Figure 2).

Cardio-vascular effort is achieved through stepping exercises, using ladder drills, or doing a slalom around gym cones by walking as fast as possible. Muscular resistance training is composed of upper limb and lower limb, flexion-extension exercises performed using body weight and light dumbbells. Balance training is composed of: (i) static exercises carried out in equilibrium of one leg on firm ground or a foam support, with eyes open or closed, and (ii) dynamic exercises, for instance, walking along a straight line or curve drawn on the ground. Coordination training consists of field exercises adapted from current research paradigms (e.g., see [41,42,43]) and previous works (e.g., see [44,45,46]). Specifically, participants perform asynchronous bimanual movements, ipsi- and contralateral arm-leg movements iso-directional or aniso-directional or four-limb coordination patterns while sitting. Dual-task training consists of counting backward, or making some easy math calculations, repeating words backward, finding words of the same family (e.g., all possible vegetables, fruits, animals), or words starting with the same syllable while performing balance, stepping, normal walking, slalom walking or, interlimb coordination tasks.

The participants will work together by pairs, distributed over the different stations, thereby allowing social interactions. Each pair starts working on one station and then goes to the next, in a predetermined order. The order of the stations will be changed every week. For each station, the two participants perform the exercises one after the other, so that working time (e.g., 45 s), plus 15 s dedicated to the stations switch of each member of the pairs, represents a rest time (75 s) for the other one. The exercise session workload is increased approximately every 2 weeks.

#### 2.6.3. Fitlight Trainer™ Circuit Training (CT-fit)

**Figure 3:**
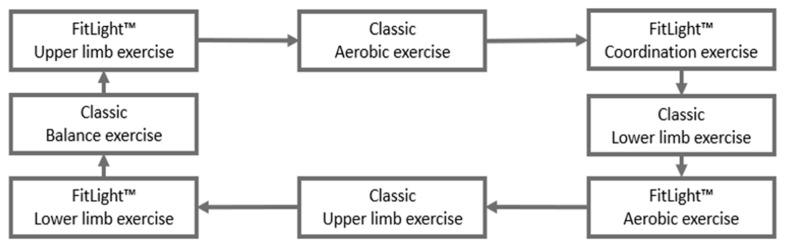
Example of the organization of the CT-fit stations.

A circuit training (CT-fit) associating cognitive and motor requirements in gamified exercises will be conceived, thanks to a fitness gaming technology (Fitlight Trainer™). The Fitlight Trainer™ is specifically designed to enhance cognitive load during physical exercises, by loading executive functions, reactivity (reaction time), movement velocity, movement accuracy, visuospatial capacities, and spatial memory while physically exercising. Practically, the Fitlight Trainer™ is a gaming technology composed of 24 different lamps that can light up in different colors and is equipped with a proximity and touch sensor wirelessly connected to a tablet. It includes wireless, RGB LED-powered lights, used as target switches, which can be turned-off by participants in a specific order, with upper and/or lower limbs, eventually requiring whole-body complex coordinated movements performed under time pressure. Thanks to the remote-control system on the tablet, various training conditions can be proposed by changing the number of colors of the target switches. Additionally, it depends also on whether the targets are placed on the ground, fixed on a wall or poles (see https://www.Fitlighttraining.com). Thus, this device allows training whole-body complex motor skills, manipulating the level of difficulty of their cognitive requirements and the intensity and duration of the physical exercises. In particular, it allows creating MwT exercises, that require the use of executive functions, visuospatial capacities, reaction time, working memory, and inhibition to succeed in the motor task. To do this, in the present experiment, the 24 lamps are divided into 4 groups of 6 lamps, each group being dedicated to one station. For each group of 6 lamps, we use a specific program proposed by the Fitlight trainer™ (the visuomotor program), which is based on random lighting of 1 to 6 possible different colors (red, blue, light blue, green, yellow, and magenta) during a fixed time period (e.g., 45 s), which represent the time allowed for completing the response. The use of different colors is a means to make the cognitive and motor component embodied one to another. For instance, by choosing two colors from 6 (e.g., red referring to the left hand and blue to the right one), the participant has to inhibit all the automatic responses when a lamp is turned on and to discriminate the color lightened and perform the corresponding motor response. Then, a number of parameters can be set, for instance, the distance of impact (i.e., how strong the impact has to be to turn it off), the time elapsing between the switch-off of one light and the random lightning of another one, and, finally, the type of lightning, static, or flashing. Notably, the program will allow each participant to personalize their movement speed and strategy to execute the cognitive-motor task properly. As with the CT-c, the CT-fit will start with a 20 min aerobic workload, then alternate stations of aerobic, muscular resistance, coordination, balance, and stepping exercises, which generally will involve the same muscle groups and physical effort of the CT-c. However, stations dedicated to dual-task training (i.e., TwM) in the CT-c will be replaced by stations dedicated to MwT training conditions (see Figure 3).

### 2.7. Data Collection and Outcome Measures

Cognitive performance, muscular force, cardiovascular and motor fitness will be assessed before and after the 8 weeks of training. Data collected with the heart rate monitors will be also analyzed, in order to estimate the average intensity of the training interventions (see [27], for a similar procedure).

### 2.8. Cognitive Assessment

Global cognitive status will be evaluated through the Montreal Cognitive Assessment (MoCA). This test is composed of different subparts and it is highly sensitive to detect MCI. Alternate versions of this test will be randomly assigned ipre- and post-test to each participant, in order to avoid test-retest effects [47]. Executive functions will be assessed through three different tests directed to: inhibition, switching, and working memory respectively. Inhibition processes will be tested with the Stroop test. Participants are seated in front of a computer and requested to indicate the color of the word appearing on the screen by pressing as quickly as possible on the corresponding key of the keyboard while inhibiting the word’s semantic. Four different colors can be presented: green, blue, red, and yellow. Thus, depending on the consistency between the semantic and the color, the condition is considered either congruent (C, e.g., the word “green” written in green) or incongruent (I, e.g., the word “green” written in red). In other trials, “neutral” words (N) are presented (N, e.g., arm, leg...), which are written in one of the different colors. After familiarization with 9 random words, 75 words will be presented for the test. Response times, that is, the time elapsing between the appearance of the word on the screen and manual pressing of the key on the keyboard, will be recorded. Each word remains present on the screen until the response is given. The mean response times (RT, in milliseconds) of the three different conditions will be calculated and compared between the others. Values above the ± 2SD of mean RT will be considered as outliers and excluded. *Switching capacities* will be evaluated through the Trail Making Test (TMT), which is composed of two parts, A and B, respectively. Part A requires participants to draw lines sequentially to connect 25 encircled numbers distributed on a sheet of paper. Part B requires participants to connect alternate numbers and letters (e.g., 1, A, 2, B, 3, C, etc.) with respect to the chronological and alphabetic order [48]. Total execution time (in milliseconds) and the number of errors will be recorded. Switching capacities are quantified by the ratio of the scores obtained in part-B divided by the scores obtained in part-A. Working memory will be assessed with a computerized test developed by the company HappyNeuron™ (Lyon, France), which is composed of two sequential parts. At first, a list of numbers will be provided to the participants via audio-speakers, and subsequently, a list of syllables. Once the full list is played, the participants are asked to repeat the last three items listened. Participants are informed that they cannot ask to listen to the track several times. The test starts with a familiarization trial. The examiner registers the correct answer and goes on with the trials. The time of completion (in seconds) and the total number of points will be recorded. Visuospatial capacities will be tested using the Rey Complex Figure copy task [49]. A complex figure comprising 18 graphic elements, a blank sheet (to copy), and a stopwatch are needed to run the test. The figure is placed in front of the participant, who must reproduce it to the best of his or her ability. The figure is exposed to the subject for a minimum of two and a half minutes and a maximum of five minutes, although it is not removed if the subject exceeds this time. The participant is allowed to copy the figure with a pen, and he/she can self-correct if he/she wants to. After the participant finishes their copy, the figure is removed. The total time required to copy the figure is measured in seconds and the quality of the response is measured for each of the 18 elements that make up the figure. A score between 0 and 2 points is given for each of these elements (maximum score of 36), depending on their accuracy, possible distortion, and location.

### 2.9. Motor Fitness Assessment

Motor fitness will be assessed through tests of balance, gait, functional mobility, flexibility, and motor coordination.

#### 2.9.1. Balance

Two tests of balance capacity will be performed using upright standing, bipedal and unipedal tasks, respectively. Bipedal upright standing will be assessed on a Force platform (AMTI, Advanced Mechanical Technology, Inc., Watertown, MA, USA). After removing their shoes, participants are helped to place their feet apart, to form an angle of 30°, in the middle of the platform. The surrounding environment is standardized, clean, and quiet, to avoid distractions. A red mark is placed on the wall in front of the participants at eye height to help them to concentrate during the eyes-opened trials. Two eyes-open and two eyes-closed trials are performed with 1 min of rest in between. For each trial, the position of the center of pressure (CoP) is recorded (sampling frequency = 100 Hz) as well as the maximum amplitude of oscillation of the center of pressure in mediolateral and anteroposterior directions (mm). In the Unipedal balance test, participants must lift one of their legs and place the foot of the free leg on the knee of the supporting leg [50] and look at a mark placed in front of them, on the wall, at eye height. Then, they are requested to keep a stable equilibrium on their preferred leg for as long as possible. The maximum trial duration is 1 min. One familiarization trial allows deciding the preferred leg to be used. Then, two trials are performed, two eyes-open, two eyes-closed and the best performance of each will be recorded. The time (in seconds) elapsing until equilibrium is lost is measured by the examiner, using the following criterion: arms must be kept along the trunk so that if the participant loses their balance and lifts them more than hip height, the examiner stops the chronometer.

#### 2.9.2. Functional Mobility

The Timed Up and Go (TUG) will be used to assess functional mobility. The participants will sit down on a chair with their feet on the floor and their hands on their legs. At the “go” signal, they have to stand up, walk 3 m at their own pace, firstly as fast as possible and after, turn around the cone, walk back, and sit down again. The performance time will start when the participant starts moving to stand up and it will end when the participant sits down again. Two trials at a self-paced velocity, and two as fast as possible are performed. The duration of the two best trials of the two different conditions will be retained [51].

#### 2.9.3. Flexibility

Hamstring’s flexibility will be tested through the Chair-sit and reach [52]. Participants are asked to sit down on a chair placed against a wall. They will be instructed to extend their preferred leg in front of their hip, with the heel on the floor and the foot dorsiflexed, and to bend the other leg so that the sole of the foot remains flat on the floor. Participants are instructed to reach down the extended leg in an attempt to touch the toes with their fingers of both hands superposed. The position has to be maintained for 3 s and 2 measurements will be performed and the best one kept. The distance (in cm) between the big toe and the hand reached point is measured with a ruler positioned parallel to the lower leg, the big toe will correspond to zero and will be compared with the recommended ranges.

#### 2.9.4. Motor Coordination

Motor coordination will be assessed using the Four-Square Stepping Test (FSST) [53]. The square is created by crossing two 90 cm long scotch tape lines in their middle point, on the floor. The four squares created are numerated with 1 in the high-left square, 2 in the high-right square, 3 in the low-right square, and 4 in the low-left square. To start the trial, the participant stands in square number 1, facing square number 2. Participants must step as fast as possible into each square in the following sequence: 2, 3, 4, 1, 4, 3, 2, and 1, which requires the participant to step forward, backward, and sideways to the right and left with the following instruction: “completing the sequence as fast as possible without touching the drawn lines, with both feet contacting the floor in each square, while facing forward during the entire sequence”. First, the sequence is demonstrated slowly to the participant by the examiner. Then, one familiarization trial is completed to ensure the participant memorized the sequence. Performance is quantified by the time taken to complete the sequence (in sec). The stopwatch starts when the first foot contacts the floor in square 2 and finishes when the last foot comes back to touch the floor in square 1. Two trials will be completed and only the best performance is considered. A trial is repeated if the participant fails to complete the sequence successfully, loses balance, or contacts a line during the sequence. Subjects who are unable to face forward during the entire sequence and needed to turn before stepping into the next square will still receive a score. Participants will be supervised by the examiner and an assistant [53].

#### 2.9.5. Gait Assessment

Gait performance will be evaluated through ten trials of single-task walking (ST) at their preferred speed, on a gait rite (GaitRite system, CIR Systems, Havertown, PA, United States). Participants will start walking and be recorded 2 m before the gait ride beginning line, and stop two meters after in order to avoid integrating acceleration and deceleration. At the end of each trial, participants have the possibility to sit down on a chair to rest, if necessary. Walking speed (cm/sec) and the size of the base of support (cm) will be measured thanks to the software of the GaitRite system.

#### 2.9.6. Dual-Task Assessment (DT)

Dual-task capacities will be evaluated with gait and cognitive tasks performed separately and simultaneously in order to determine the cost of dual-tasking on each function. Three single tasks (ST, two orals, one walking) and two dual-task (DT) conditions, consisting of walking while performing, orally, a cognitive task (i.e., the Trail Making Test, TMT) will be performed. The full procedure is inspired by those proposed by Ho and colleagues [54]. The oral Trail Making Test (OTMT) [55] is composed of two parts: the OTMT part-A (OTMT-A) and the OTMT part-B (OTMT-B). In part-A, participants will be asked to count as fast as possible with the lowest possible number of errors from 1 to 25. In part-B, participants are asked to alternate numbers and letters, starting with 1-A-2-B-3-C…, until the examiner asks to stop. The test is stopped when the participants have given 25 responses. The time of completion and the number of correct responses is recorded. The single gait walking task (ST- described in the gait assessment paragraph, see above) is chosen as a motor task. In the DT conditions, the DT-OTMT-A is performed at first. Participants are asked to perform the OTMT-A task, (procedure described above) while walking at their preferred speed. Then, the OTMT-B task is performed while walking at their preferred speed. Time of completion and the number of correct (number and letters) alternation is recorded (a shift in the sequence of the answers is only considered as an error). By dividing the values recorded for cognitive or gait performances during ST-OTMT-B and DT-OTMT-B by those obtained in the single tasks ST-OTMT-A and the DT-OTMT-A, respectively, a score representing cognitive flexibility is obtained and used as an outcome variable. In order to calculate the DT cost on processing speed, response times measured in DT-OTMT-A are divided by response times measured during the OTMT-A. To calculate the DT cost on mental switching, response times in the DT-TMT-B are divided by response times in DT-TMT-A. The calculation of the cost of cognitive tasks on gait will be done by dividing the score related to walking speed or size of the base of support recorded during the DT walking trial with the same variable recorded in the ST trail.

### 2.10. Physical Fitness

Muscular strength and cardiovascular capacities will be tested to assess physical fitness.

#### 2.10.1. Muscular Strength

The maximal handgrip strength (in Kg) will be assessed using a hand dynamometer (JAMAR^®^ hand dynamometer, Chicago, IL, USA), as a proxy measure of the general strength (i.e., sarcopenia) [56]. Participants sit on a chair without armrests clenching the hand around a dynamometric handle, they put their hand on their leg to relax the shoulder and to avoid the elbow’s movements. One practice trial is allowed and then, three attempts for each side alternated, with a 30 s rest between, are performed. Only the best of the three trials of each hand is considered for analysis. The strength and fatigue tolerance of the lower limbs is assessed with the Timed-stands test [57]. Participants sit on a chair without armrests placed against a wall to prevent it from moving during the test. The test starts with the participant seated in the middle of the chair, back straight, feet on the floor approximately shoulder-width apart. Arms are crossed at the wrists and held against the chest. At the signal, the participant has to stand up fully (body erect and straight) and then go back to the initial seated position 10 times as fast as possible. The participants are encouraged to complete the repetitions without using their arms. The participant is clearly instructed to be fully seated between each stand. Two repetitions to assess and fully understand the movement are permitted before the test session [57]. The time to complete the 10 repetitions will be recorded.

#### 2.10.2. Cardiovascular Capacities

The 10 m incremental shuttle walking test (SWT) will be assessed to determine the functional capacity of older individuals [58]. It is performed in a 10-m course, identified by two cones placed 0.5 m from each endpoint, with an initial speed of 0.5 m/s, increasing 0.17 m/s every minute. In the present study, the original protocol (12 levels) is slightly modified by extending the test up to 15 levels of 1 min, since only healthy subjects are involved. The walking speed prescribed by a metronome: brief periodic beeps indicate when the participants should be turning around the cone. The test is terminated when participants are unable to cross the cones by the time the signal occurred on three consecutive occasions (i.e., are >0.5 m from the cone), or when they feel too breathless and/or fatigued to continue and/or show signs of physical discomfort (symptoms as dyspnea, dizziness, and vertigo) and/or no longer wish to continue. Verbal instructions are given by examiners when the participants fall behind on the required walking/running speed, providing the participant an opportunity to catch up in the next one. The last completed speed level is considered as the maximal walking speed (Vmax, in km/h). Participants subsequently rate their perceived exertion by using the Borg Scale. The post-exercise HRmax and RPE will be also recorded. Moreover, HR will be measured after 3 min rest, to ensure that participants’ HR relax to pre-exercise levels.

### 2.11. Data Management

The included participants will be registered with an identification number, and all the data collected will be anonymized. Data will be collected and saved on a separate hard disk and treated anonymously or, for each identification code, a logbook will be started in which test results from baseline and post-intervention assessments will be collected as well as the documentation on each training session. In terms of data quality, a trained project-assistant will verify the data entries and check the digitalized version after all training and assessments are finished.

### 2.12. Sample Size

The sample size calculation was performed using G. Power 3.1.9.7 (Kiel, Germany) [59]. In order to detect relevant between groups differences and in the changes from pre- to post-intervention, we calculated a sample size sufficient to detect medium effect sizes. Therefore, f = 0.25, α = 0.05, *p* = 0.80, were chosen to favor clinically significant effect sizes. To test the main hypotheses of the present study, 42 participants, are required. In order to compensate for possible dropouts, 3 more participants will be included. The three groups are composed of 15 participants each.

### 2.13. Statistical Analysis

Data will be analyzed with SPSS (SPSS Inc., Chicago, IL, USA). The statistical analysis will allow us to determine the effects of the three different training programs on the cognitive, motor, and physical variables. The analysis will include subjects who completed all the pre- and post-tests. Data of the pre-test and post-test will be presented using descriptive statistics: mean, standard deviation, and percentage to describe the participants’ characteristics and performance on assessments. Comparisons within and between groups at baseline and post-intervention will be done using repeated measures ANOVA, and Newman-Keuls tests as post hoc analyses. The global Anova carried out between groups will allow testing the existence of a training effect (pre/post) for the different groups. Training is expected to improve performance for the three groups for both cognitive and physical variables.

With respect to the post-comparisons, we predict that no statistical differences should be observed between groups in the pre-test, while differences should be observed in the post-test. Specifically, post-hoc comparisons of the performance in the post-test should allow determining whether, as predicted, CT-fit training leads to greater benefits than the two other training modalities.

Baseline differences between groups will be carried out by observing the % of progress (pre/post-training) in each group, in some relevant measures. Adherence to programs will be measured by the absence rate (in %).

## 3. Discussion

This experiment aims at comparing, in older adults, the effects of three different training protocols, which differ in their cognitive and motor content. In the following, we present the different hypotheses and the planned related analyses.

### 3.1. Differences in the Effectiveness of the Three Training Protocols on Cognitive, Physical, and Motor Capacities

In accordance with the literature [60,61], global cognitive performance is expected to be enhanced after training in all three groups. However, it is expected that the CT-c and CT-fit protocols will lead to larger improvements in EF than NW. This should result from the combination of the effects of coordination training and aerobic exercise on cognition [10,15] that is allowed by the synergy between facilitation and guidance effects that occur at the brain level [35,62,63]. Moreover, a strong hypothesis is that CT-fit will be a more effective training protocol to improve EFs [64]. It should be the case since the MwT training conditions used in the CT-fit require the implementation of cognition in action, which is closer to real-life situations than TwM task conditions used in the CT-c. Additionally, according to Herold and colleagues [30], MwT training has a higher potential than TwM training to stimulate brain plasticity, due to the multiple sensory systems activated and involved [30]. In summary, one could expect that the CT-fit will show larger benefits for executive function as compared to the other groups. Nevertheless, the two other forms of training are also predicted to lead to improvements in EFs. In addition, due to the presence of specific DT exercises in the CT-c, participants in this training protocol will show better dual-task performance in the related tests. The question remains, however, of the possible benefits of the other protocols on DT performance. This issue will be addressed. Finally, visuospatial capacities are expected to be more improved by the CTs, especially by the CT-fit due to the embodied characteristics, than in NW. Indeed, it has been previously demonstrated that coordination training specifically impacts visuospatial capacities [12,14]. With respect to physical fitness, reflected by cardiovascular capacities, in accordance with Temprado and colleagues’ [27] study, it is expected that all three groups will improve their cardiovascular capacities. This could occur if the mean level of effort, indexed by mean heart rate calculated during the different sessions for the three training programs, is roughly equivalent [27]. If not the case, a difference could be observed between the three groups in the relative test. In case of differences between the three groups, we will analyze heart rate and data from the Borg scale to determine whether one of three training protocols was (at least perceived) of higher intensity, relative to the others. Regarding muscular capacities, it is expected that the two CTs will show a significant improvement in the related tests, larger than those observed in NW. Motor fitness is expected to be impacted by the three training programs, as previously observed by Temprado and collaborators [27]. However, one expects that the two CTs should be more effective than NW in this respect. Indeed, presumably, NW requires the repetition of automated arm-leg coordination (stabilization by quantitative repetition), while the CT programs allow developing whole-body and multi-limb coordination through a variety of different exercises, which may be learned over the different sessions and could allow qualitative incorporation of new behavioral patterns in the repertoire of existing ones. Thus, NW and CTs are hypothesized to develop motor fitness and coordination capacities [27], while differently, due to the respective part of repetitive practice of automated movements, variety and novelty of the to be learned coordinated movements. In addition, one expects to observe an improvement in walking speed (cm/sec), in the single task in the NW group relative to the CT groups. Additionally, a decrease in the size of the base of support (cm) is expected more for the CTs groups, proving the effectiveness of the specific balance training proposed in these training groups. Finally, as in our previous study, the percentage of responders (i.e., participants who progressed in the exercise under interest) will be also assessed to check whether it is higher for some protocols, though differentially for specific tests. In the lack of related studies available in the literature, no hypothesis is made in this respect but these analyses could help to interpret eventual differences of performance between groups and tests.

### 3.2. Strength and Weakness of the Present Study

Several important theoretical and practical issues are addressed in the present study. The first one lies in the comparison between circuit training protocols based on the association of aerobic exercise, muscular resistance training, and either TwM or MwT situations (i.e., CT-c and CT-fit, respectively). If our hypotheses are confirmed, this study will be the first one to show that MwT circuit training implemented via fitness gaming technologies are more effective multi-domain training protocols in older adults. The effects of the NW training protocol are also of great practical interest. Indeed, the results of our previous study [27] suggested that NW is a cheap and very effective way to improve physical, motor, and cognitive capacities in older adults. Notably, the sanitary situation (i.e., Covid-19) could make it difficult to regroup people over 65 years in closed spaces and then, to carry out the study. Additionally, some participants might be discouraged by the use of new technology in the CT-Fit protocol. However, nowadays they are becoming way more familiar with them. Thus, we do not anticipate that the use of technology will be a problem. On the contrary, it could be a motivating factor. Thus, the superiority of the CT-fit training over the more classical training conditions would allow us to conclude that this type of technology augmented training is among the most effective training to improve cognition and physical functions. These types of exercises would therefore be recommended as an optimal solution for the prevention of older adults’ cognitive and physical decline.

### 3.3. Benefits and Risk for Participants

The evaluation is designed to be adapted and safe for a population of healthy older adults. The risks are essentially associated with the physical activities involved in the three intervention groups. However, the risk is lowered by a medical exam prior to training, and the supervision of skilled coaches, ensuring that the training is safe for all the participants.

## 4. Conclusions

The validation of the hypothesis concerning the effectiveness of the CT-fit on the improvement of cognition compared to the CT-c and NW should confirm the effectiveness of incorporating the fitness game. Clearly, the other two training programs should show an improvement in the more stimulated skills (for instance, CT-c should show better performance on DT performance). In addition, this new theoretical framework, based on the incorporation of cognitive stimulation into complex goal-oriented actions, implemented by fitness game technology, will create a new line of reasoning within the vast literature on exergames, for the current time focused on the entertainment component of exergames rather than the creation of related theoretical frameworks. In fact, it would show the need for a specific and controlled physical effort combined with cognitive stimulation, in order to make the game of fitness effective.

## Figures and Tables

**Table 1:** Inclusion and exclusion criteria.

Inclusion Criteria	Exclusion Criteria
Participant ≥65 ≤80, good health condition, sedentary behavior Passed maximal cycle-ergometer effort test	Cognition < 26/30 on the MMSE IPAQ = highly active Sign of cognitive impairment Uncontrolled psychiatric or cardiovascular affections Uncorrected earing and/or visual impairment Psychotropic or bradicardizing medical treatments Participants familiar with the Fitlight Trainer™ methods

## Data Availability

The data presented in this study will be available on request from the corresponding author. The data are not publicly available, as they have not yet been collected.
